# A Comparison Study of Tie Non-response Treatments in Social Networks Analysis

**DOI:** 10.3389/fpsyg.2018.02766

**Published:** 2019-01-15

**Authors:** Feifei Huang, Minqiang Zhang, Yan Li

**Affiliations:** ^1^School of Psychology, South China Normal University, Guangzhou, China; ^2^Guangdong Key Laboratory of Mental Health and Cognitive Science, South China Normal University, Guangzhou, China

**Keywords:** social networks, tie non-response, complete-case approach, unconditional mean imputation, reconstruction, multiple imputation

## Abstract

Analysis of social network data often faces the problem of tie non-response. Recent studies show that the results of social network analyses can be severely biased if tie non-response was ignored. To overcome the problems created by tie non-response, several treatments were proposed in the literature: complete-case approach, unconditional mean imputation, reconstruction, and multiple imputation. In this paper we assessed the impact of tie non-response on social network analysis and investigated the performance of four treatments to handle tie non-response. The simulation results showed that ignoring tie non-response data in network analysis could underestimate the degree and centralization of social networks depending on the types of network and the proportion of missing ties. We also found that unconditional mean imputation was the best tie non-response treatment. Multiple imputation could successfully correct for tie non-response in a few specific situations. Complete case approach and reconstruction, however, were not recommended. We advocate the importance of further research to better understand consequences of tie non-response in social networks analysis and to provide statistical guidance to researchers to tackle this problem in the field.

## Introduction

Social network analysis focuses on relationships among entities, and on the pattern and implications of these relationships, which has attracted considerable curiosity and interest from the social science community in recent years (Wasserman and Faust, 1994). In social network applications, the nodes represent actors, and the ties represent a specific relationship between actors (Handcock and Gile, 2010). However, the complexity of social network survey is more likely to generate incomplete data, which means that some actors or ties are missing from the dataset (Kossinets, 2006). Researchers often encounter the situation that response rates vary from 65 to 90% (Stork and Richards, 1992; Borgatti and Molina, 2003; Costenbader and Valente, 2003; Kossinets, 2006; Huisman, 2009). Besides, several studies showed that missing data have a negative effect on structural properties of networks. For instance, the strength of relationships and clustering coefficients are likely to be underestimated, and centrality and degree measures will become unstable (Borgatti and Molina, 2003; Costenbader and Valente, 2003; Kossinets, 2006; Huisman, 2009). It is worth briefly noting that social network analysis is especially sensitive to missing data.

Non-respondents create significant and potentially insidious problems for network analysis (Robins et al., 2004). Non-response includes complete actor non-response and tie non-response (Žnidaršič et al., 2012). Actor non-response occurs when actors are absent and all data from them are missing. Tie non-response occurs when actors participate in the survey but the data on particular ties are absent. But the distinction between ties that are missing and ties are really not present cannot be made. Non-response has particular negative effects on those multiple interaction situation networks. For example, if an actor fails to respond or whose ties are missing in an affiliation network, we may have a limited capacity to describe the network context. Besides, those actors with whom they interact will cause large amount of missing data (Robins et al., 2004; Kossinets, 2006). Several studies found that the ignorance of non-response in network research have a negative effect on network mapping and estimating structural network properties (Borgatti and Molina, 2003; Kossinets, 2006). More researchers begin to focus on proposing some ways to ameliorate the problem of non-response in social network analysis (Daniel, 1975; Stork and Richards, 1992; Kossinets, 2006; Huisman and Steglich, 2008; Žnidaršič et al., 2012).

Although non-responses in social network analysis may now be receiving more sustained attention over the last two decades, methods for effectively dealing with non-response continue to require further development (Robins et al., 2004; Kossinets, 2006). Huisman (2009) investigated four simple imputation procedures to handle non-response and found that simple imputations can only successfully correct for non-response in a few specific situations. Koskinen et al. (2013) introduced Bayesian imputation procedures based on ERGM for partially observed network data, missing ties, attributes, and actors. The model-based ERGM approach is able to model social network data and does not require the independence assumption implicit in logistic regression (Robins et al., 2004; Gile and Handcock, 2006; Handcock and Gile, 2010), and it is the most sophisticated imputation procedures. In general, the network literature provides little guidance on how to deal with missing data when there is non-response (Robins et al., 2004). Our concern here is to investigate the effects of tie non-response treatments and to provide practical and simpler options for researchers to deal with tie non-response problems.

This paper presents the results of a simulation study that addresses these two issues. First, the effect of tie non-response on the structure of a network was investigated. Second, the performance of four approaches to treat the tie non-response was inspected by studying the effect of treatments on two network measures. The rest of this paper is organized as follows. Firstly, we focuses on four treatments in social network analysis. Secondly, the design of the simulations is presented. Then, we presents the results with respect to three missingness mechanisms. Finally, we presents a discussion of the results and some general recommendations.

## Non-Response Treatments

### Complete-Case Approach

The complete-case approach removes both the non-respondents' incoming and outgoing ties, which is known as “listwise deletion.” Taking an example of an emotional relationship network into consideration shown in Table 1, we can note that the network having three non-respondents A, D and F reports no outing tie (denoted with label N). The complete-case approach is based on a smaller network of completely observed actors as shown in Table 2, because the approach removes all ties between non-respondents and respondents (Huisman and Steglich, 2008; Žnidaršič et al., 2012).

**Table 1:** Emotional relationship network with three non-respondents (A, D, and F) provide no outing tie.

	**A**	**B**	**C**	**D**	**E**	**F**	**G**
A		N	N	N	N	N	N
B	1		1	0	1	0	0
C	1	1		0	1	1	1
D	N	N	N		N	N	N
E	1	1	1	0		1	0
F	N	N	N	N	N		N
G	1	0	1	1	1	0	

**Table 2:** The complete-case approach.

	**B**	**C**	**E**	**G**
B		1	1	0
C	1		1	1
E	1	1		0
G	0	1	1	

The complete-case approach is also known as a weighting method, which discards the information of non-respondents and equally weights the completely observed actors and non-respondents. The method is simple, but it is only applicable to some patterns of missing data (Little and Rubin, 2014). Researchers also found that the complete-case method might be valid only when non-respondents are missing completely at random (Schafer and Graham, 2002).

### Unconditional Mean Imputation

Unconditional mean imputation is a simple imputation procedure proposed by Schafer and Graham (2002), which is replacing each missing tie with the mean of the observed ties. In the social networks analysis, there are three ways to impute the unconditional mean (Huisman, 2009): (1) impute the average number of relations which is the density of the network; (2) impute the incoming relations of an actor; (3) impute the outgoing relations of an actor. For example, in the binary networks, the unconditional mean is equal to the network density, and this procedure imputes zeros in sparse networks and ones in dense networks. When coping with missing network data, Žnidaršič et al. (2012) noted that this imputation procedure requires some threshold. An application of imputing the unconditional mean procedure is given by Huisman (2009), who used 0.5 as the threshold in his research.

The procedure of imputing the unconditional mean is one of the popular approaches used to handle missing data in social networks analysis because of simplicity (Gabbay and Zuckerman, 1998; Schafer and Graham, 2002; Huisman, 2009). But Huisman (2009) argues that this simple imputation procedure may produce biased estimates and underestimate some uncertainty levels.

### Reconstruction

Reconstruction of the missing part of the network using observed incoming relations of missing actors is suggested by Stork and Richards (1992). When applying reconstruction to missing data in social network analysis, there are two criteria should be met (Stork and Richards, 1992): one is the similar pattern between non-respondents and observed actors, the other is the information from observed actors should be reliable. According to types of social network, there are two different ways to use the reconstruction procedure (Stork and Richards, 1992; Huisman, 2009; Žnidaršič et al., 2012): (1) in the directed network, all missing ties of non-respondents *i* are replaced with the observed incoming relation of the opposite tie from respondents *j*:$x_{ij}^{imp}=x_{ji}$, (2) in the undirected network, both the completely observed ties between respondents and partially observed ties between respondents and non-respondents are used. Taking an example of a friendship network into consideration shown in Table 3, we can note that the network having three non-respondents A, D and F reports no outing tie (denoted with label N). The reconstruction approach is shown in Table 4.

**Table 3:** Friendship network with three non-respondents (A, D, and F) provide no outing tie.

	**A**	**B**	**C**	**D**	**E**	**F**	**G**
A		N	N	N	N	N	N
B	1		1	0	1	0	0
C	1	1		0	1	1	1
D	N	N	N		N	N	N
E	1	1	1	0		1	0
F	N	N	N	N	N		N
G	1	0	1	1	1	0	

**Table 4:** The reconstruction approach.

	**A**	**B**	**C**	**D**	**E**	**F**	**G**
A		1	1	N	1	N	1
B	1		1	0	1	0	1
C	1	1		0	1	1	1
D	N	0	0		0	N	1
E	1	1	1	0		1	1
F	N	0	1	N	1		0
G	1	0	1	1	1	0	

The reconstruction procedure allows researchers to maximize the available information of social networks (Neal, 2008). More and more researchers are in favor of using this method to cope with the problem of missing data (Gabbay and Zuckerman, 1998; Huisman and Steglich, 2008; Neal, 2008; Huisman, 2009; Alexey et al., 2011; Žnidaršič et al., 2012). However, reconstruction of ties between two non-respondents is impossible, additional imputations are required to reconstruct the network.

### Multiple Imputation

Multiple imputation, proposed by Rubin (1987), in which each missing value is replaced by a list of *m*>1 simulated values drawn from their conditional distribution. In social network analysis, multiple imputation is also an attractive method to solve the problem of missing data (Durrant, 2009; Handcock and Gile, 2010; Lee et al., 2016; Wang et al., 2016). The general procedure of multiple imputation is:

- impute *m* (*m* > 1) plausible values to replace each non-response or missing tie by imputation models. The imputation models vary according to types of data and missingness. For discrete data, the logistic regression imputation model is widely used (Brand, 1999).
- analyze each of the *m* data sets with a complete-data method, then each parameter has *m* estimates and standard errors.
- combine the results of *m* analyses by Rubin's rules (1987) to obtain overall estimates and standard errors.

Compared to single imputation, multiple imputation can reflect the uncertainty of missing values through the variation among *m* imputations. And the method can solve problems caused by some simple imputation methods (Huisman, 2009). However, multiple imputation is computationally complicated and require a lot of imputations to produce the complete data sets (Schafer and Olsen, 1998).

## Simulation Study

In order to compare the effects of tie non-response treatments in social network analysis, a simulation was performed. The process of the simulation study was as follows:

- generate a complete network and analyze the network data;
- create non-response by deleting a proportion of ties;
- apply four different tie non-response treatments to deal with the incomplete network and generate a completed network;
- re-analyze the completed network data and evaluate the performance of four tie non-response treatments.

To study the performance of item non-response treatments, three missing mechanisms were analyzed separately, using the same design. For each missing mechanism, there were three independent variables used to generate complete data and missing data: two types of networks, three sample sizes and three proportions of missing ties, resulting in 2 × 3 × 3 = 18 cells. And each cell was repeated 500 times for each condition. The simulation study was conducted using R 3.3.1 software, and we used the “mice” package in R 3.3.1 to deal with tie non-response for the multiple imputation method.

### Generating Network Data

The network used in this study was based on real data of a friendship network from the *Teenage Health and Lifestyle* study provided with the StOCNET software (Boer et al., 2006). Data are available from the StOCNET software (http://www.stats.ox.ac.uk/~snijders/siena_links.htm). The network data was a subset of the friendship network, which consisted of 50 actors and directed relations between them, as used in Pearson and West (2003), Steglich et al. (2006), Huisman and Steglich (2008), and Huisman (2009). The friendship data were assessed by a name generator that each actor could nominate up to six best friends. Alcohol consumption was recorded by a 5-point frequency questionnaire ranging from 1 (“I don't drink”) to 5 (“more than once a week”).

There were two types of network used in the simulation study: the original directed network and the undirected network created by replacing ties of each pair of actors with their maximum value of ties in the original network. The density of the undirected network was 0.066 and the density of the directed network was 0.047.

In order to consider the equal interval between each sample size, three sample sizes were used in the simulation study: 50, 150, and 250. The sample size of 50 was based on real data of a friendship network from the *Teenage Health and Lifestyle* study. The sample sizes of 150 and 250 were created by the *as.network* (Butts, 2008) function of package *SNA* in *R* 3.3.1 based on the density of the undirected network and the directed network, which was 0.066 and 0.047, respectively.

### Creating Tie Non-response

Three different tie non-response mechanisms were created according to three missingness mechanisms defined by Rubin (1976): Missing Completely At Random (MCAR), Missing At Random (MAR), and Missing Not At Random (MNAR). The probability of a tie was missing based on regimes (Huisman and Steglich, 2008; Huisman, 2009): (1) MCAR, ties were missing completely at random, (2) MAR, the probability of missing ties was proportional to 1/(*alcohol score*)^2^, (3) MNAR, the probability of missing ties was proportional to 1/(*outdegree*+1)^2^. The data are MAR because the alcohol score is completely observed for all actors, and the data are MNAR because the missingness is related to a network characteristic determined from the complete data set. Both the MAR and MNAR mechanisms are such that higher scores result in small tie non-response probabilities. Three proportions of missing ties used in the simulation study were 0.05, 0.1, and 0.25.

### Performance of Tie Non-response Treatments

To investigate the precision and accuracy of the estimated network measures (degree centrality and centralization) for four tie non-response treatments, the bias (as shown in Equation 1) for the network measures were analyzed across conditions, where ${\overset{⌢}{\theta }}_{i}$, θ represented the final network measures estimate and known network measures, respectively, and *r* was the total number of repeated times in each condition.

(1) $$\begin{array}{l} Bias\left(\overset{⌢}{\theta }\right)=\overset{r}{\underset{i=1}{\sum }}\left({\overset{⌢}{\theta }}_{i}-\theta \right)/r \end{array}$$

To compare the performance of four treatments, the centrality and the centralization were analyzed across conditions. For centrality, we used the degree centrality to describe the network positions (as shown in Equation 2). For centralization, we calculated network centralization to describe the network structure (as shown in Equation 3).

(2) $$\begin{array}{l} {C^{\prime }}_{D}\left(p_{k}\right)=\frac{\overset{n}{\underset{i=1}{\sum }}a\left(p_{i},p_{k}\right)}{n-1} \end{array}$$

(3) $$\begin{array}{l} C=\frac{\overset{n}{\underset{i=1}{\sum }}\left(C_{\max}-{C^{\prime }}_{D}\right)}{\max\overset{n}{\underset{i=1}{\sum }}\left[\left(C_{\max}-{C^{\prime }}_{D}\right)\right]} \end{array}$$

In Formulas (2), and (3), *a*(*p*_*i*_, *p*_*k*_)represented the number of ties between pair of points, $C_{D}^{\prime }$, *C*_max_ represented the centrality defined above and the largest value of $C_{D}^{\prime }$ for any point in the network, and $\max\overset{n}{\underset{i=1}{\sum }}\left[\left(C_{\max}-{C^{\prime }}_{D}\right)\right]$ represented the maximum possible sum of differences in centrality for a network of n points (Freeman, 1978).

## Results

**Figure 1:**
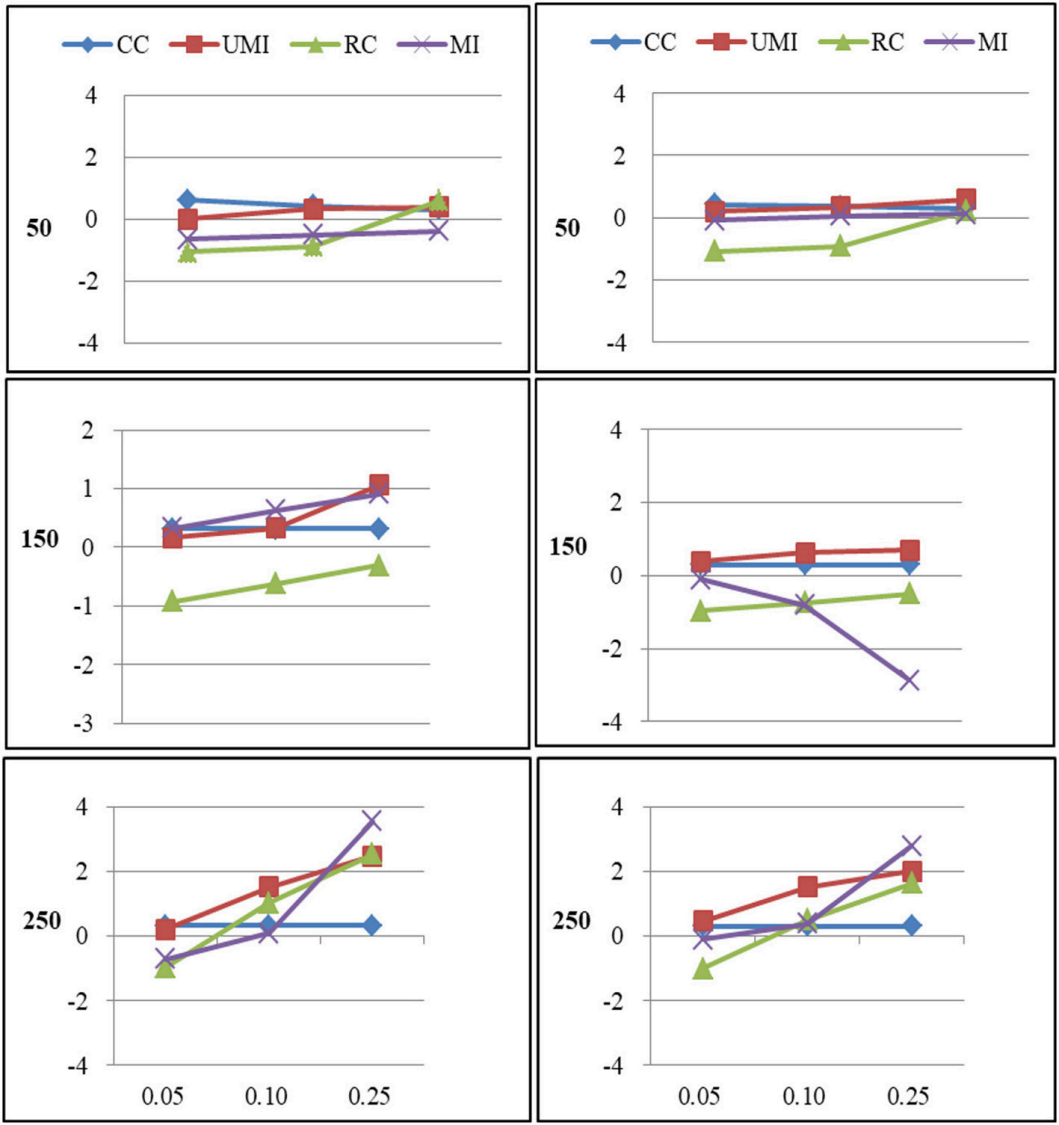
Standardized Bias of degree of four non-response treatments under the MCAR mechanism. From left to right: directed network and undirected network. From top to bottom: sample sizes of 50, 150, and 250 on the y axis, and proportion missing data on the x axis. Within each panel four different lines represent the non-response treatments: CC (Complete Case approach), UMI (Unconditional Mean Imputation), RC (Reconstruction), and MI (Multiple Imputation).

**Figure 2:**
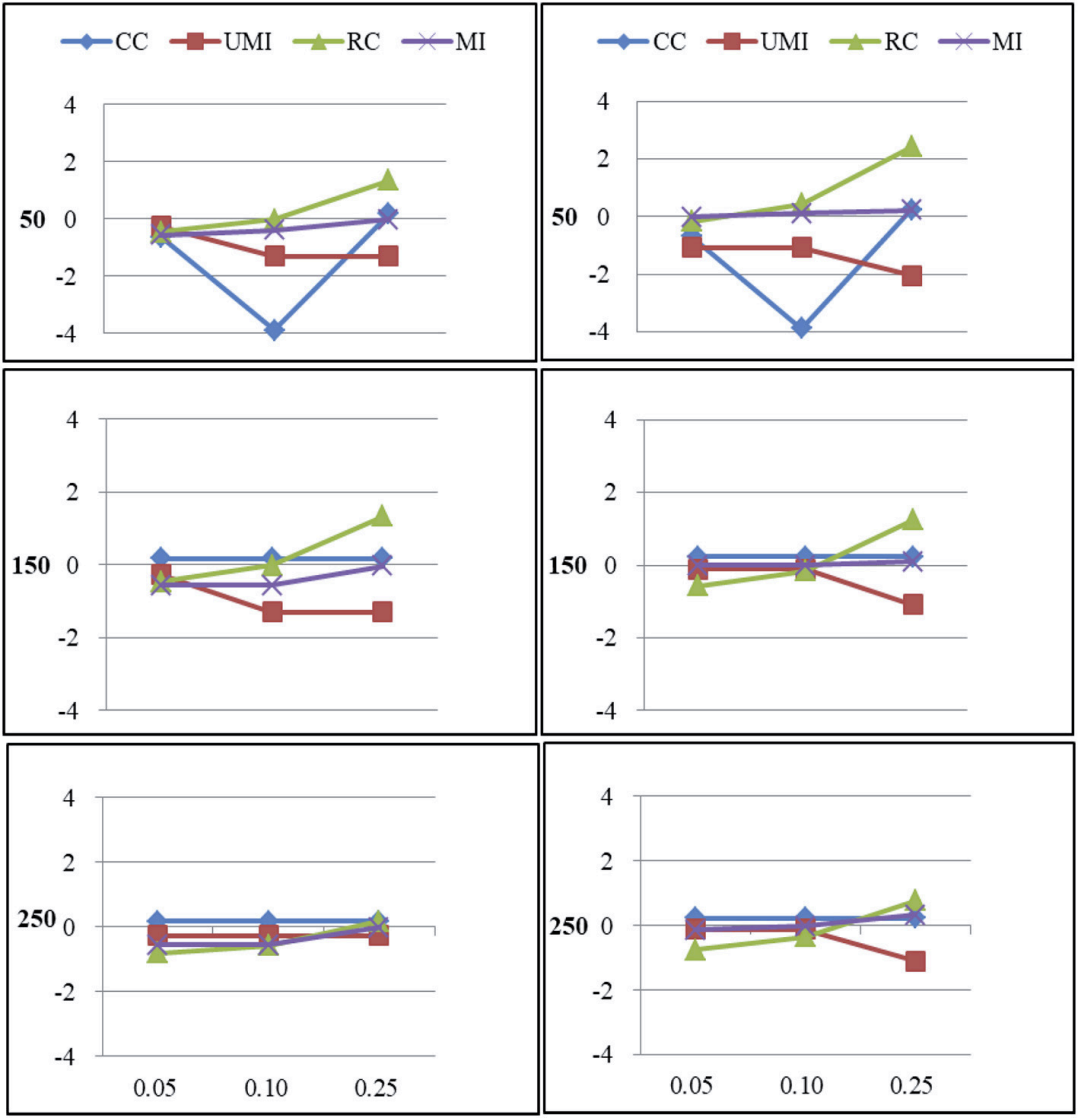
Standardized Bias of centralization of four non-response treatments under the MCAR mechanism. From left to right: directed network and undirected network. From top to bottom: sample sizes of 50, 150, and 250 on the y axis, and proportion missing data on the x axis. Within each panel four different lines represent the non-response treatments: CC (Complete Case approach), UMI (Unconditional Mean Imputation), RC (Reconstruction), and MI (Multiple Imputation).

**Figure 3:**
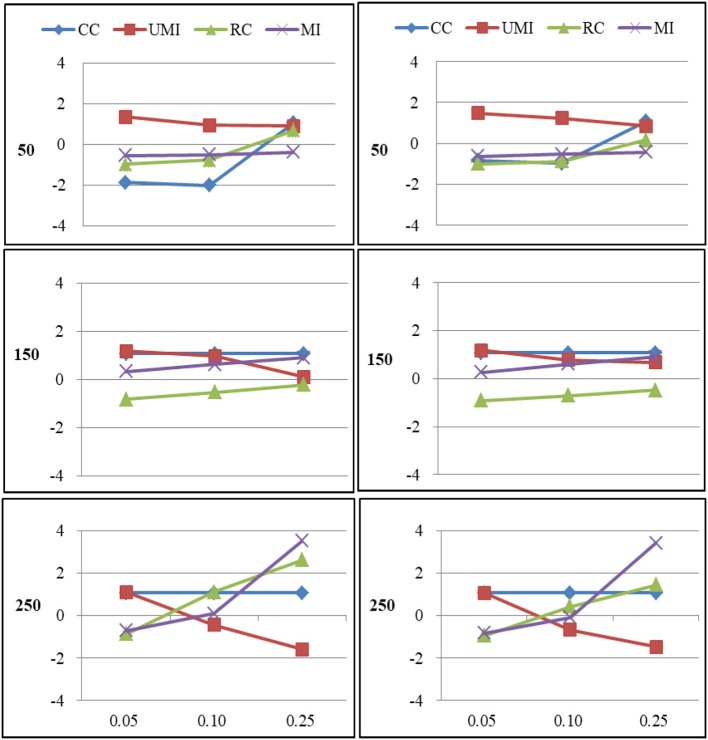
Standardized Bias of degree of four non-response treatments under the MAR mechanism. From left to right: directed network and undirected network. From top to bottom: sample sizes of 50, 150, and 250 on the y axis, and proportion missing data on the x axis. Within each panel four different lines represent the non-response treatments: CC (Complete Case approach), UMI (Unconditional Mean Imputation), RC (Reconstruction), and MI (Multiple Imputation).

**Figure 4:**
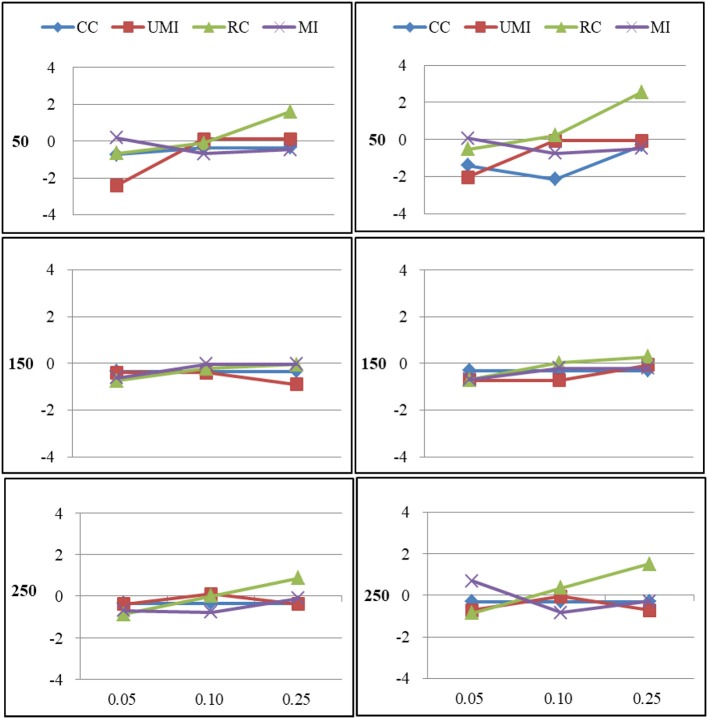
Standardized Bias of centralization of four non-response treatments under the MAR mechanism. From left to right: directed network and undirected network. From top to bottom: sample sizes of 50, 150, and 250 on the y axis, and proportion missing data on the x axis. Within each panel four different lines represent the non-response treatments: CC (Complete Case approach), UMI (Unconditional Mean Imputation), RC (Reconstruction), and MI (Multiple Imputation).

**Figure 5:**
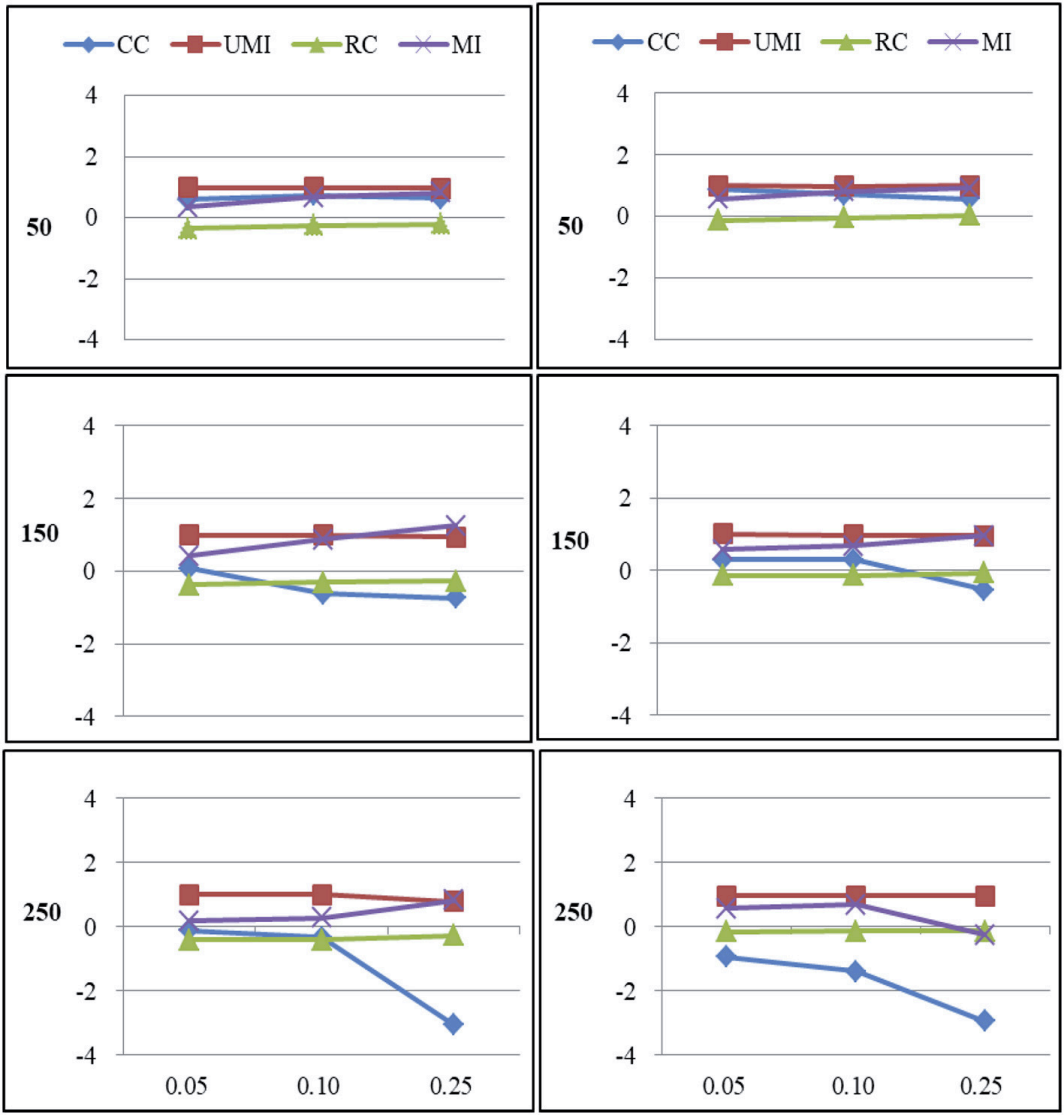
Standardized Bias of degree of four non-response treatments under the MNAR mechanism. From left to right: directed network and undirected network. From top to bottom: sample sizes of 50, 150, and 250 on the y axis, and proportion missing data on the x axis. Within each panel four different lines represent the non-response treatments: CC (Complete Case approach), UMI (Unconditional Mean Imputation), RC (Reconstruction), and MI (Multiple Imputation).

**Figure 6:**
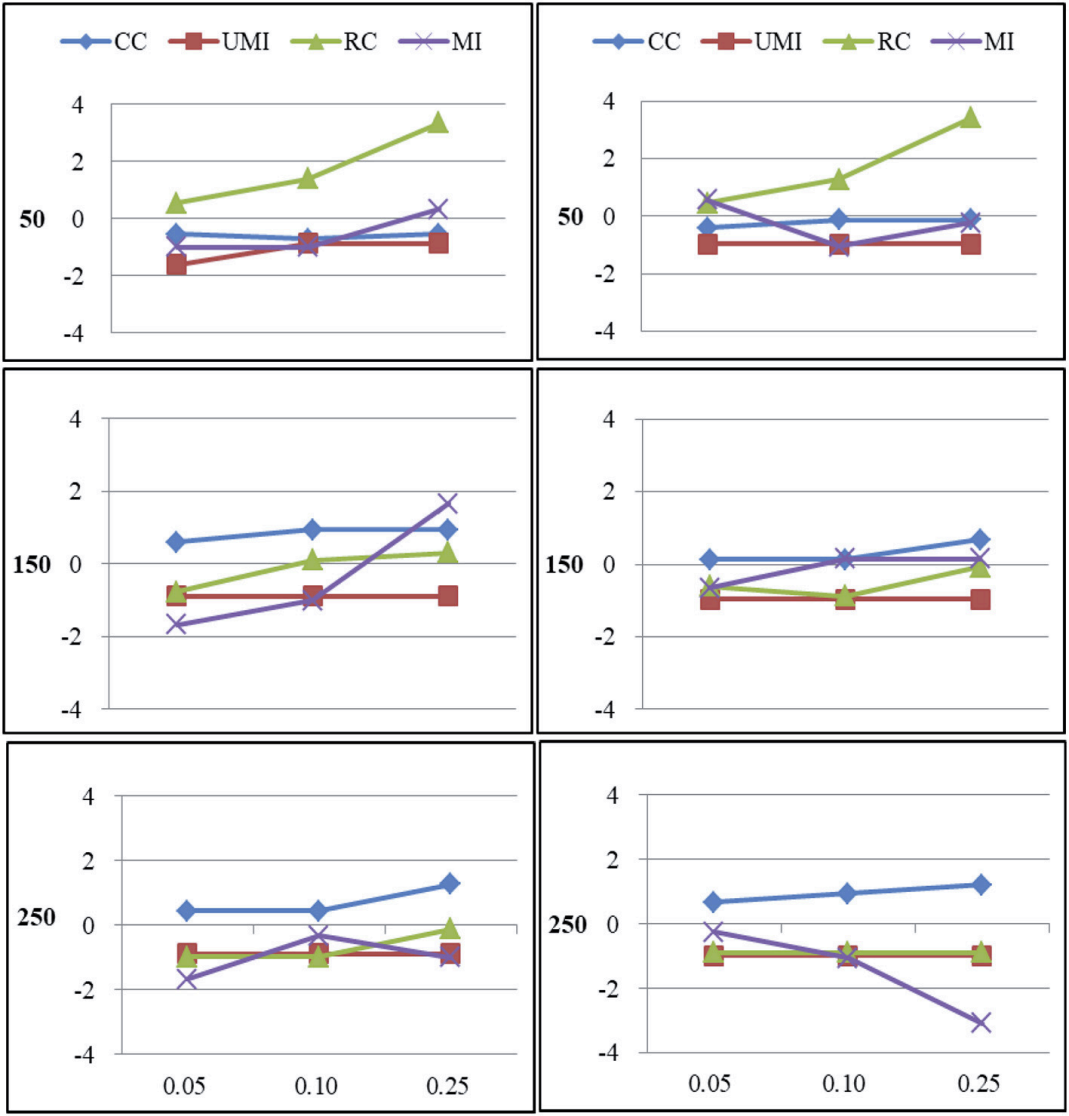
Standardized Bias of centralization of four non-response treatments under the MNAR mechanism. From left to right: directed network and undirected network. From top to bottom: sample sizes of 50, 150, and 250 on the y axis, and proportion missing data on the x axis. Within each panel four different lines represent the non-response treatments: CC (Complete Case approach), UMI (Unconditional Mean Imputation), RC (Reconstruction), and MI (Multiple Imputation).

The results of the simulations were shown according to three missingness mechanisms. Figures 1–6 presented the Bias for four treatments under each combination of sample sizes, proportions of missing ties and types of networks. Within each plot, lines for four non-response treatments CC (Complete Case approach), UMI (Unconditional Mean Imputation), RE (Reconstruction) and MI (Multiple Imputation) were shown. Figures on the left side showed the results for directed networks, figures on the right side for undirected networks. The sample sizes were presented from top to bottom on the y axis of the figures, on the x axis corresponded to the proportions of missing ties.

### Missing Completely at Random

For degree there were large effects of proportions of missing ties and sample sizes (as shown in Figure 1). Of these four treatments, the results for reconstruction were the worst because it had the largest bias in most of plots and the final network measure estimates from using reconstruction were larger than the known network measures. There were two treatments that performed quite well according to the bias: unconditional mean imputation and multiple imputation. Of these two treatments, the former performed slightly better. The complete-case approach was not applicable when the proportion of missing ties was high. So results from using this method were unacceptable. In general, as the proportion of missing ties increased the bias for four treatments grew. Compared to undirected networks, the bias was larger in the case of directed networks. When we had 50 and 150 actors, results from all treatments were acceptable. However, for 250 actors, differences in the results emerged for four treatments. The result was stable for unconditional mean imputation and multiple imputation. For reconstruction, the increase was so large that the results were unacceptable.

For centralization there were large effects of proportions of missing ties (as shown in Figure 2). In general, as the proportion of missing ties increased the bias for four treatments grew. Compared to undirected networks, the bias was slightly larger in the case of directed networks. However, for smaller sample size differences in the results emerged for four treatments. The result was stable for unconditional mean imputation and multiple imputation. For reconstruction, the increase was so large that the results were unacceptable.

### Missing at Random

For degree there were large effects of proportions of missing ties and sample sizes (as shown in Figure 3). Of these four treatments, the results for reconstruction were the worst because it had the largest bias in most of plots and the final network measure estimates from using reconstruction were larger than the known network measures. There were two treatments that performed quite well according to the bias: unconditional mean imputation and multiple imputation. Of these two treatments, the former performed slightly better. The complete-case approach was not applicable when the proportion of missing ties was high. So results from using this method were unacceptable. In general, as the proportion of missing ties increased, biases for four treatments grew. Compared to undirected networks, the bias was larger in the case of directed networks. When we had 150 actors, the results from all treatments were acceptable. However, for 50 and 250 actors, differences in the results emerged for four treatments. The result was stable for unconditional mean imputation and multiple imputation. For reconstruction, the increase was so large that the results were unacceptable.

For centralization there were large effects of proportions of missing ties (as shown in Figure 4). In general, as the proportion of missing ties increased, biases for four treatments grew. The results for all treatments were nearly the same in both types of networks. When we had 150 actors, the results from four treatments were acceptable. However, for 50 and 250 actors, differences in the results emerged for four treatments. The result was stable for unconditional mean imputation and multiple imputation. For reconstruction, the increase was so large that the results were unacceptable.

### Missing Not at Random

For degree there were large effects of proportions of missing ties and sample sizes (as shown in Figure 5). Of these four treatments, results for the complete-case approach were the worst because it had the largest bias in most of plots and the final network measure estimates from using the complete-case approach were larger than the known network measures. There were three treatments that performed quite well according to the bias: unconditional mean imputation, reconstruction and multiple imputation. In general, as the proportion of missing ties increased, biases for four treatments grew. Compared to undirected networks, the bias was larger in the case of directed networks. When we had 50 actors, the results from all treatments were acceptable. However, for 150 and 250 actors, differences in the results emerged for four treatments. The result was stable for unconditional mean imputation, reconstruction and multiple imputation. For the complete-case approach, the increase was so large that the results were unacceptable.

For centralization there were large effects of proportions of missing ties and sample sizes (as shown in Figure 6). In general, as the proportion of missing ties increased, biases for four treatments grew. The results for all treatments were nearly the same in both types of network. When we had 150 and 250 actors, the results from all treatments were acceptable. However, for 50 actors, differences in the results emerged for four treatments. Results were stable for the complete-case approach, unconditional mean imputation and multiple imputation. Of these three treatments, the first treatment performed slightly worse. For reconstruction, the increase was largest that the results were unacceptable.

## Discussion

Tie non-response has a large negative effect on analyzing social network. In this paper, we conducted a simulation study to investigate the effect of four treatments to treat the tie non-response. The simulations were based on an empirical friendship network, and tie non-response was created using different types of networks, sample sizes and proportions of missing ties.

The simulations showed that ignoring tie non-response data in network analysis could underestimate the degree and centralization of social networks depending on the types of network and the proportion of missing ties. Comparing directed and undirected networks (Figures 1–6), the biases were somewhat larger in directed networks. The direction of tie non-response effects on the descriptive analyses were generally the same. For centralization biases were larger for both types of networks. Results found by Smith and Moody (2013) was similar for centrality measures in those situations where directed networks were less robust than undirected networks. Consistent with previous studies, estimates were worse with more missing data (Huisman and Steglich, 2008; Huisman, 2009; Žnidaršič et al., 2012; Smith and Moody, 2013; Smith et al., 2017). Besides, we found that smaller networks were more robust to missing data. Previous studies had demonstrated that smaller networks were more centralized (Borgatti et al., 2006; Smith and Moody, 2013). Further, results of the present study revealed that the missingness mechanisms had a smaller effect, where generally the non-random missingness mechanism leads to the largest biases in estimating degree-related statistics.

In the simulations, the unconditional mean imputation was the best tie non-response treatment. For small to large networks, it gave better results than the other three treatments both in directed and undirected networks. And even for larger amounts of missing ties, the unconditional mean imputation was the recommended treatment when calculating descriptive statistics. Besides, results of the study showed that the multiple imputation also produced good results in most situations. But the treatment needed more computational time than the unconditional mean imputation. The other two treatments, reconstruction and the complete-case approach, generally resulted in more bias. Reconstruction was expected to correct the effects of tie non-response in some situations. However, it often failed when both ties in a dyad were missing. This result was also found by Huisman (2009). Moreover, it should be noted that the performance of the complete-case approach depended on the missingness mechanisms. For random missingness mechanisms, the complete-case approach broke down because of it was not applicable when the proportion of missing ties was high. For non-random missingness mechanism, the treatment was able to correct the bias, except for degree in case of medium to high proportions of missing ties.

Based on the study, the following recommendations can be given:

- The use of the unconditional mean imputation is suggested to deal with the tie non-response in the social network analysis. Multiple imputation can also be recommended if the proportion of missing ties is low, or when the network is relatively small.
- Do not use the reconstruction treatment or complete-case approach if researchers encounter the situation when actors participate in the survey but the data on particular ties are absent in the social network analysis.

This paper aims to highlight the problem of tie non-response in social network analysis. However, this study has some major limitations. Firstly, networks with other structures would reveal different performances, which would make generalizing the results of the study to denser network difficult (Huisman, 2009). Secondly, results of the study revealed that performances of some treatments did rather well on small (50) and large (250) sample sizes but worse on medium (150). It will be useful to expanding the sizes of the networks that we considered. Furthermore, with sparse networks, we have shown that tie non-response is a serious problem in social network analysis. It is likely that we need to explore more methods to deal with the problem. For example, the “link prediction” techniques would be also a useful tie non-response treatment. Until these extensions are made, we can make generalization to situations that are explored.

## Author Contributions

FH designed the study, conducted the simulation study, and drafted the manuscript. MZ participated in designing the study and revised the manuscript. YL conducted the literature review. All authors read and approved the final manuscript.

### Conflict of Interest Statement

The authors declare that the research was conducted in the absence of any commercial or financial relationships that could be construed as a potential conflict of interest.
